# Intermittent theta burst stimulation (iTBS) and inhibitory control training to reduce binge eating: study protocol for a randomized controlled trial (Be-Nemoic)

**DOI:** 10.3389/fpsyg.2026.1677814

**Published:** 2026-02-02

**Authors:** Marta Becerra-Losada, Andrea Bernat-Villena, Francisco Javier Pérez-Comino, Luz Stella Algarra-López, Raquel Vilar-López, Alfonso Caracuel

**Affiliations:** Mind, Brain and Behaviour Research Centre (CIMCYC), University of Granada, Granada, Spain

**Keywords:** binge eating, dorsolateral prefrontal cortex, inhibitory control training, iTBS, neuromodulation, ventromedial prefrontal cortex

## Abstract

**Background:**

Binge eating is a complex and often underdetected condition characterized by recurrent episodes of excessive food intake accompanied by a perceived loss of control, leading to significant psychological and health consequences. Although pharmacological, psychological, and nutritional treatments are available, their effectiveness remains limited—possibly due to an insufficient understanding of the underlying cognitive and neurobiological mechanisms. Impulsivity, particularly food-specific impulsivity, has been identified as a key factor in binge eating, with inhibitory control deficits associated with increased cravings, maladaptive eating behaviors, and impaired decision-making. Recent studies suggest that inhibitory control training and non-invasive brain stimulation—especially intermittent theta burst stimulation (iTBS) targeting prefrontal regions such as the dorsolateral (dlPFC) or ventromedial prefrontal cortex (vmPFC)—may offer promising therapeutic effects. However, the combined use of cognitive and neuromodulatory interventions has been scarcely investigated. This study aims to determine the efficacy of iTBS combined with inhibitory control training in individuals with binge eating.

**Methods and analysis:**

In this double-blind, randomized, controlled trial with parallel groups, 150 individuals with binge eating will be allocated to one of three groups. All participants will undergo inhibitory control training, differing only in the stimulation site: (1) active iTBS of the dlPFC, (2) active iTBS of the vmPFC, or (3) control site vertex stimulation. The interventions will consist of ten sessions over 2 weeks. Primary outcomes will be binge eating symptoms and food craving. Secondary outcomes will include changes in brain activation and connectivity (via fMRI), cognitive functioning, eating behavior, and biological markers. Assessments will be conducted pre-treatment, post-treatment, and at 3-month follow-up. Health service utilization will also be collected to evaluate cost-effectiveness and cost-utility.

**Discussion:**

The results of this study will contribute to the evidence base for combined cognitive and neuromodulatory interventions aimed at improving eating behavior in individuals with binge eating.

**Trial registration:**

The study was registered at http://www.clinicalTrials.gov, number NCT06649994.

## 1 Introduction

Binge eating (BE) is characterized by the consumption of substantial amounts of food within a short period of time, accompanied by a pervasive sense of loss of control, psychological distress and guilt (Mohajan, 2023; Myers and Wiman, 2014). It was only introduced as an independent disorder in the Fifth Edition of the Diagnostic and Statistical Manual of Mental Disorders (DSM-5) (American Psychiatric Association, 2013) and the Eleventh Revision of the International Classification of Diseases (ICD-11) (Claudino et al., 2019). To be considered a disorder, BE episodes must occur at least once a week for 3 months. An estimated 1.5% of women and 0.3% of men worldwide meet the DSM-5 criteria for BE disorder (Erskine and Whiteford, 2018). However, it is important to note that many individuals frequently engage in BE without meeting full diagnostic criteria (Striegel-Moore et al., 2000), or they may not seek professional help due to the shame and guilt associated with the behavior (Preti et al., 2009). Thus, estimating the prevalence of BE is challenging and the condition is frequently under-detected (Keski-Rahkonen, 2021). BE shows high comorbidity with other psychological disorders, particularly mood, anxiety and substance abuse disorders (Kowalewska et al., 2024), and has significant consequences for overall health (Flayelle and Lannoy, 2021).

Several pharmacological (Himmelstein et al., 2017), psychological and nutritional (Mars et al., 2025) treatments are available for BE, however, their efficacy remains limited (Linardon et al., 2017). One likely contributing factor is the limited understanding of the mediating and predictive variables underlying this behavior. In this context, impulsivity has been investigated as a key factor in understanding and addressing BE (Davis, 2013). Impulsivity is a multidimensional construct reflecting poor reward-related decision-making and a tendency to engage in rash or reward-seeking behaviors (Dawe and Loxton, 2004). Difficulties in impulse control are associated with several psychopathologies (Bari and Robbins, 2013), and some evidence suggests that people with BE show greater impulsivity in general (Yan et al., 2022), and specifically in relation to food (Schag et al., 2013a,b). Moreover, heightened impulsivity in individuals with BE has been linked to greater severity of eating disorder psychopathology, more pronounced depressive symptoms, and higher rates of comorbidity (Boswell and Grilo, 2021). According to some models of impulsivity, people with BE may experience stronger motivation to approach palatable foods and are more likely to act impulsively on this motivation (Dawe and Loxton, 2004), thereby compromising their decision making. This pattern would result in an increase in craving for food (i.e., an intense desire to consume highly rewarding food) and impair the ability to inhibit or control eating behavior (Gullo et al., 2014; Ng and Davis, 2013). Therefore, the development and evaluation of impulse-focused interventions may represent a promising therapeutic avenue (Giel et al., 2017). Following on from the above, one of the most relevant targets for intervention is inhibitory control (Treasure et al., 2015; Smith et al., 2020; Kessler et al., 2016; Sarlo et al., 2025).

Some interventions targeting food-related impulsivity have yielded better outcomes in reducing BE than psychotherapeutic approaches that do not address this component (Ferrer-García et al., 2017; Preuss et al., 2017). In particular, food-specific inhibitory control training—a bottom-up approach aimed at modulating automatic responses to appetitive food cues via motor inhibition (Jones et al., 2016)—has shown promising effects (Allom et al., 2016). Inhibitory control training interventions using food imagery within Go/NoGo paradigms over a 4-week period have demonstrated greater reductions in BE frequency, valuation of high energy-dense foods, food approach behaviors, anxiety, and depression, compared to non-food-specific versions of the same training (Chami et al., 2022). In line with these findings, a study evaluating the *FoodT* mobile application in patients with BE disorder or bulimia nervosa—delivering food-related inhibitory control training via food Go/NoGo training—reported improvements in psychopathological symptoms, valuation of high energy-dense foods, food addiction, and lack of premeditation after 4 weeks of daily training (Keeler et al., 2022). Effective eating self-control balances responses to food cues with top-down inhibition. Food-specific Go/NoGo paradigms capture this balance more directly than abstract tasks and underline the role of inhibitory control when highly palatable cues are present (Bianco et al., 2023).

On the other hand, multiple brain alterations associated with BE behaviors have been identified. Some studies report hyperactivation in limbic reward regions (Yu et al., 2022) and hypoactivation in frontal regions involved in inhibitory control (Donnelly et al., 2018). Neuroimaging findings from food-related inhibitory control tasks consistently show reduced activation in the dorsolateral prefrontal cortex (dlPFC), ventromedial prefrontal cortex (vmPFC) and inferior frontal gyrus (Lavagnino et al., 2016; Saruco and Pleger, 2021; Murray et al., 2023; Haynos et al., 2021; Veit et al., 2021). These results suggest that individuals with BE have hypoactivation in frontal regions, particularly within the prefrontal cortex, which is considered a key area for self-regulation and inhibitory control (Pasquale et al., 2024). Accordingly, non-invasive neuromodulation targeting these areas has emerged as a promising intervention to reduce BE (Wu et al., 2024).

Non-invasive brain stimulation refers to techniques that modulate brain and network activity via non-surgical procedures. Repetitive transcranial magnetic stimulation (rTMS) is one of the most commonly used methods in this context (Wu et al., 2024). Although studies using multisession rTMS for the treatment of BE are scarce, a single-case study reported significant improvements in BE frequency and psychopathological symptoms after a multisession rTMS intervention targeting the left dlPFC (Pires Baczynski et al., 2014). Likewise, (Pánek and Donchev, 2025) reported decreases in BE symptoms, food cravings, and body weight following six sessions of dlPFC-targeted rTMS. Consistently, Maranhão et al. (2025) found a significantly greater reduction in BE episodes after 20 sessions of neuronavigated rTMS of dlPFC compared to control site vertex stimulation.

A form of rTMS that present promising advantages is intermittent theta burst stimulation (iTBS), as it enables shorter sessions durations, which is beneficial from a cost-benefit perspective (Rachid, 2017). Importantly, theta rhythms facilitate long-term potentiation (Larson et al., 1986), allowing iTBS to induce faster and longer-lasting effects on synaptic plasticity. To date, only two studies have applied iTBS to the dlPFC in individuals with disordered eating behaviors, with promising results. The first study, a case report, described that after 18 sessions of iTBS applied to the left dlPFC—using 30 Hz triplet bursts repeated at 5 Hz (2sec. on, 12.3sec. off; 600 pulses per session) at 80% of the motor threshold—binge-eating episodes completely stopped in two women (Sciortino et al., 2021). The second study, conducted in 22 women with dysregulated eating behaviors, showed that a single session of dlPFC stimulation significantly reduced drive for thinness and body dissatisfaction—key risk factors for eating disorders (Barone et al., 2023). The stimulation protocol involved 50 Hz bursts, each consisting of 3 pulses, delivered in 10 bursts per 8-second cycle, with 20 cycles in total, resulting in 600 pulses overall. Stimulation intensity was set at 30% of the maximum stimulator output and kept consistent across participants, as previous evidence suggested that adjusting intensity to individual motor thresholds does not necessarily enhance the effects (Kaminski et al., 2011). This is the study we have selected to specify the stimulation parameters for the present protocol.

In short, the prefrontal cortex has been the primary target for neuromodulation research in eating disorders due to its accessibility and its role in inhibitory control (Gallop et al., 2022). Specifically, non-invasive brain stimulation studies have focused on the dlPFC (Lee et al., 2017), and to date, no studies have targeted other areas such as the vmPFC in the treatment of BE, despite its research interest (Wu et al., 2024).

In summary, both cognitive training and neurostimulation of brain areas implicated in BE show promise as therapeutic interventions, but their combined effects and the impact of repeated sessions remain insufficiently explored (Wu et al., 2024; Gouveia et al., 2021). In particular, the effectiveness of inhibitory control training may be enhanced when combined with neurostimulation (Woods et al., 2016).

Furthermore, previous literature remains scarce, and the need for methodological improvements has been underscored, such as more comparable study designs, including harmonized neurostimulation protocols, placebo compared groups and the use of neuronavigation for high-precision targeting (Herwig et al., 2003). In particular, neuronavigation informed by individual structural MRI (Liu et al., 2025)is essential for head-to-head site comparisons, maximizing targeting accuracy, reproducibility, and the internal validity of the stimulation procedure (Fitzgerald et al., 2009). On the other hand, pairing iTBS with a concurrent therapeutic component is important to engage the same processes during stimulation (Wu et al., 2024).

Nevertheless, this hypothesis remains to be tested, as do the brain, cognitive and behavioral changes that may result from these interventions, as well as the distinct effects of neuronavigated stimulation of distinct brain areas involved in BE compared to control site stimulation. The protocol of this trial was designed to overcome these gaps. The main objective of the present study is to determine the effects of neuromodulation using iTBS targeting the left dlPFC and in left vmPFC in combination with inhibitory control training, aiming to induce changes at the brain, cognitive, behavioral, emotional, and biological levels in individuals with binge eating. Active stimulation will be compared to its control site vertex counterpart. The specific aims of the study are: (i) To determine the effectiveness of iTBS targeting the dlPFC in combination with inhibitory control training for the treatment of BE behaviors, assessing improvements in BE symptoms, food valuation and intake, cognitive functioning biological measures; (ii) To determine the effectiveness of iTBS targeting the vmPFC in combination with inhibitory control training for the treatment of BE, using the same outcome parameters; (iii) To characterize the brain changes associated with the interventions (iTBS of the dlPFC vs. iTBS of the vmPFC, both combined with inhibitory control training) using fMRI; (iv) To examine the relationship between biological markers obtained in blood, saliva, urine and feces—as well as candidate genes—and neuropsychological and emotional variables (depression, anxiety, stress, emotional regulation, emotional eating, craving, inhibition, food valuation, delay of gratification, impulsivity, working memory, cognitive flexibility and decision making), as well as neuroimaging indices (brain activation, gray and white matter volume and connectivity); (v) To perform an economic evaluation of the cost-effectiveness and cost-utility of the interventions for individuals with BE and to analyze the budgetary impact of their implementation within the public health system.

We hypothesize that both interventions are expected to reduce BE symptoms, cravings, and unhealthy food valuation, while also producing changes in brain connectivity and improvements in eating behavior, inhibitory control and cognitive functioning. Additionally, the interventions are expected to yield benefits in terms of cost-effectiveness and cost-utility.

## 2 Methods

### 2.1 Experimental design, setting, and dates

The study is a double-blind, randomized, controlled trial with three parallel groups, conducted at the Mind, Brain, and Behavior Research Centre (CIMCYC) at the University of Granada (Granada, Spain). Part of the interaction will take place on site, while other components will be delivered online through services contracted by the University of Granada, including Google Meet (videoconferencing for presentation and evaluation sessions), Lime Survey, and the Millisecond Test Library (used for administering and recording responses to evaluation instruments during assessment sessions). In addition, participants will receive messages on their mobile phones as part of the intervention. The study was registered at http://www.clinicalTrials.gov number NCT06649994 in October 2024, and adheres to the SPIRIT reporting guidelines (Chan et al., 2013). All items from the World Health Organization Trial Registration Data Set are summarized in Table 1, in accordance with SPIRIT guidelines. Data collection began in April 2024, and it is expected to be completed by March 31, 2026. No financial compensation will be provided for participation in the study.

**Table 1:** Items from the World Health Organization Trial Registration Data Set.

**Section/item**	**Information**
Primary registry and trial identifying number	http://www.clinicalTrials.gov, NCT06649994.
Date of registration in primary registry	21, October 2024.
Secondary identifying numbers	P21_00776
Source(s) of monetary or material support	Junta de Andalucía Mind, Brain and Behaviour Research Centre (CIMCYC), University of Granada.
Primary sponsor	Prof. Vilar-López, R. and Prof. Caracuel, A.
Secondary sponsor (s)	Becerra-Losada, M., Bernat-Villena, A., Pérez-Comino, F.J., and Algarra-López, L.S.
Contact for public queries	Prof. Alfonso Caracuel, acaracuel@ugr.es
Contact for scientific queries	Prof. Raquel Vilar-López, rvilar@ugr.es
Public title	Be-Nemoic study.
Scientific title	Intermittent theta burst stimulation (iTBS) and inhibitory control training to reduce binge eating: study protocol for a randomized controlled trial (Be-Nemoic)
Countries of recruitment	Spain.
Health condition(s) or problem(s) studied	To study the efficacy of neuromodulation with iTBS to the binge eating treatment in combination with inhibitory control training.
Intervention(s)	1. Combined intervention: active stimulation of the dlPFC with iTBS and inhibitory control training 2. Combined intervention: active stimulation of the vmPFC with iTBS and inhibitory control training 2. Control intervention: control site vertex iTBS and inhibitory control training.
Key inclusion and exclusion criteria	People between 18 and 60 years old with 2 binge eating episodes per month to 8 per week and no contraindication for performing fMRI (pregnancy, metal implants, etc.) or iTBS.
Study type	Interventional Allocation: randomized Intervention model: parallel assignment Masking: double Primary purpose: treatment
Date of first enrolment	November 2024.
Target sample size	150.
Recruitment status	Recruiting.
Primary outcome(s)	Outcome name: Binge eating and food craving. Method of measurement: the Binge Eating Scale (BES) and the Food Cravings Questionnaire (FCQ-t-r) Timepoint: pre-treatment, post-treatment and follow-up.
Key secondary outcomes	Changes in neuroimaging measures Timepoint: pre-treatment and post-treatment Changes in eating behavior Timepoint: pre-treatment, post-treatment and follow-up.
Ethics review	Approved by Research Ethics Committees of Andalusia on April 16, 2024.
Completion date	March 31, 2026.

The SPIRIT reporting guidelines recommend that these items be included in trial protocols to provide a brief, structured overview of a trial.

### 2.2 Sample size calculation

The sample size was calculated using the G^*^Power 3.1 tool G-Power v3.1.9.7 (Heinrich Heine University Düsseldorf, Düsseldorf, Germany). To do so, we relied on the few studies that explored the effects of inhibitory control training (Allom et al., 2016; Keeler et al., 2022) or iTBS (Barone et al., 2023) to treat binge eating or dysregulated eating behaviors. Effect sizes were small for body dissatisfaction (0.27), drive for thinness (0.30), food addiction (0.46), or health behaviors (0.378), and medium for eating psychopathology (0.57). Thus, considering a small effect size for conducting ANOVAs (f = 0.15), the minimum recommended sample size to reach a power of 0.95 and alpha level of 0.05 to calculate the interaction model of the three groups and three repeated measures was 141 (47 participants per group). We opted for a conservative *N* = 150 (*n* = 50 per group). At least *n* = 30 per group will conduct the fMRI to explore brain changes.

### 2.3 Recruitment, participants, eligibility criteria, data collection, management, and analysis

Participants will be recruited through social and mass media, the project website (trainep.ugr.es), posters and the Granada University's official communication channels. Eligible candidates will be individuals aged 18 to 60 years, with proficiency in Spanish and a BMI between 20 and 39.9 kg/m^2^. Individuals with mild to moderate binge eating symptoms will be included, excluding those with binge eating frequencies classified severe. Specifically, participants reporting at least two binge eating episodes per month and up to seven episodes per week will be selected. Participants must have access to at least two electronic devices (e.g., tablet, computer or smartphone) to attend the online meetings and perform the online assessments.

Although heterogeneity may complicate interpretation, the inclusion range (BMI 20–39.9 kg/m^2^) is consistent with evidence showing that binge-eating disorder occurs across weight categories (Carbone et al., 2023; O'Loghlen et al., 2025; Barnes and Lawson, 2024) and with methodological guidance emphasizing that restrictive eligibility reduces external validity and recruitment (e.g., PRECIS-2, CONSORT extensions; (Loudon et al., 2015) Potential variability will be addressed by recording illness duration, age, and education, reporting baseline comparability, and performing adjusted, sensitivity, and subgroup analyses. On the other hand, an upper age limit of 60 years minimizes age-related variance in executive and inhibitory control (Lemire et al., 2024). Overall, this strategy is consistent with the broader binge-eating literature and supports both methodological rigor and clinical relevance. All candidates will be screened for medical and psychological disorders and excluded if they meet any of the following criteria: (i) traumatic, metabolic, or endocrine disorders; (ii) cardiovascular or other conditions that preclude physical exercise; (iii) psychopathological disorders or severe symptoms with suicidal ideation, as assessed by the Depression Anxiety and Stress Scale-21 (DASS-21) (Daza et al., 2002); (iv) eating disorders other than binge eating, as assessed by the Questionnaire on Eating and Weight Patterns-5 (QEWP-5) (Yanovski et al., 2015); (v) contraindications for undergoing functional magnetic resonance imaging (e.g., pregnancy, metal implants) or iTBS (e.g., tinnitus, dizziness, surgical interventions, neurological conditions, or use of drugs affecting the central nervous system); (vi) current use of pharmacological or other treatments for weight loss; (vii) being a candidate for bariatric surgery; (viii) weight loss greater than 5% in the 3 months prior to the intervention.

Participants (*N* = 150) will be randomly assigned, using computer-generated random codes, to one of three groups: (i) Group 1: active iTBS targeting the left dlPFC combined with inhibitory control training (*n* = 50); (ii) Group 2: active iTBS targeting the left vmPFC combined with inhibitory control training (*n* = 50); Group 3: active control group receiving control site vertex iTBS combined with inhibitory control training (*n* = 50). Participants may withdraw from the trial at any time; however, under no circumstances the intervention allocation will be modified.

The psychologist conducting the assessments (screening, evaluation sessions, and follow-ups) will remain blinded to group allocation throughout the entire study. Further, all participants will be blinded to their assigned condition. Additionally, the researchers performing the statistical analyses will be also blinded to group allocation using coded intervention labels. The psychologist responsible for delivering the interventions and the nurse assisting with the stimulation sessions will be the only unblinded personnel and will generate the allocation. Therefore, no additional unblinding procedure will be required.

The informed consent form is the only document containing non-anonymized information. It will be collected in paper format and securely stored under lock and key. The database will contain no personally identifiable information and will be created and stored on a computer without internet access.

### 2.4 Outcome measures

Assessments will be conducted at three time points: pre-treatment, post-treatment, and 3-month follow-up.

#### 2.4.1 Main outcome measure

*Change score across time in binge eating and food craving measured at pre-treatment (week 2), post-treatment (week 5) and follow-up (week 17)*.

a) **Binge eating symptoms**. The Binge Eating Scale (BES) (Gormally et al., 1982) is a self-administered questionnaire consisting of 16 items: eight assessing behavioral manifestations of binge eating (e.g., eating fast or consuming large amounts of food), and eight assessing associated thoughts and emotions (e.g., fear of not stopping eating). Each item is scored from 0 to 3 (0 = no symptom severity, 3 = severe symptomatology).
b) **Binge eating symptoms**. Questionnaire on Eating and Weight Patterns-5 (QEWP-5) (Yanovski et al., 2015) is a self-administered assessment instrument designed to identify possible cases of BE disorder according to DSM-5 criteria. This instrument explores the frequency, duration and characteristics of binge eating, discomfort and control of episodes.
c) **Food Craving**. The Food Cravings Questionnaire (FCQ-t-r) (Innamorati et al., 2015) will be administered to obtain a total score indicative of food craving as both state and trait.

#### 2.4.2 . Secondary outcomes

*Change across time in neuroimaging measures at pre-treatment (week 2) and post-treatment (week 5) assessments*.

a) **Resting-State Brain Connectivity**. During resting-state acquisition, participants will be instructed to remain still, with eyes closed and as relaxed as possible, attempting not to focus on any specific thoughts for 6 min. These scans will be used to examine resting-state connectivity of brain networks. Specifically, seed-based functional connectivity analysis will be performed taking the stimulated region (dlPFC or vmPFC) as the seed. This will allow assessment of functional connectivity changes in these regions and across the whole brain.
b) **White matter integrity**. Diffusion tensor imaging (DTI) will be acquired during a 10-min scan while participants remain still. This technique provides indirect measures of white matter architecture and connectivity.
c) **Functional task: fMRI Food Specific Go/NoGo Task** [based on (Lawrence et al., 2015)]. Participants will be instructed to respond as quickly and accurately as possible by pressing a button on a response device located inside the scanner when a green circle is presented (Go signal), and to withhold their response when a red circle is presented (No-Go signal). Images of high- and low-calorie foods will be presented, as well as neutral non-food images (items of daily life). This inhibitory control task uses the same stimuli as the intervention training. In this evaluation version, 50% of images of healthy food, unhealthy food and neutral will be presented under “Go” conditions, and 50% of each stimulus type (healthy food, unhealthy food and neutral) will be presented under “No-Go” conditions. Brain activation will be analyzed by comparing responses to Go vs. No-Go stimuli and to food vs. non-food stimuli.
d) **Functional task: Food decision making** [based on (Neveu et al., 2018)]. This task comprises three blocks inside the scanner: healthiness, palatability and decision making. Participants will rate 50 foods using a 5-point Likert scale for perceived healthiness (Block 1) and palatability (Block 2). In Block 3, participants will choose between a reference food—selected algorithmically from the neutral-rated items in Blocks 1 and 2—and an alternative food. These forced binary choices are designed to induce cognitive conflict when the reference food is more palatable than the alternative, or the alternative is healthier than the reference food. In the cognitive conflict studies, selection of the healthier food is defined as a controlled choice, and selection of the tastier food as an uncontrolled choice. Brain activation will be compared between the controlled and uncontrolled choice conditions.

#### 2.4.3 Predictive and mediating/moderating variables

*Change scores across time in eating behavior at pre-treatment (week 2), post-treatment (week 5) and follow-up (week 17)*.

a) **Eating behavior:** Dietary intake will be assessed using the Food frequency questionnaire (CFA) (Vioque et al., 2019) which includes 52 items. Participants will report the quantities of all foods and beverages consumed over the last year (pre-treatment), the past 2 weeks (post-treatment) and the past 3 months (follow-up). These data will be converted into estimates of total caloric intake, as well as calories derived from fats, carbohydrates, and sugars.

*Changes in emotional symptoms and emotional eating at pre-treatment (week 2), post-treatment (week 6) and follow-up (week 18)*.

a) **Stress and Anxiety. The Depression Anxiety Stress Scale-21**: (DASS-21) (Daza et al., 2002) is a dimensional self-report instrument designed to assess negative emotional states. For this study, only the stress and anxiety subscales will be used, each comprising seven items. Participants rate the extent to which each item applied to them over the past week, using a 4-point Likert scale.
b) **Depression symptoms**. The Beck Depression Inventory (BDI-II) (Sanz et al., 2003) is a 21-item self-report measure assessing the severity of depressive symptoms. It has been widely used in clinical and treatment outcome research.
c) **Emotion Regulation**. Emotion Regulation Questionnaire (ERQ) (Cabello et al., 2013) is a 10-items self-report scale that evaluates two emotion regulation strategies: cognitive reappraisal and expressive suppression.
d) **Emotional eating**. The Coping subscale of the Palatable Eating Motives Scale (PEMS) (Burgess et al., 2014) consist of 4 items assessing the tendency to consume palatable foods in response to negative emotions.
e) **Reward-related eating**. The Reward-Based Eating Scale (RED) (Mason et al., 2018) is a 13-item scale that assesses preoccupation with food, perceived loss of control over intake, and difficulties achieving satiety.
f) **Non homeostatic eating**. The Dutch Eating Behaviour Questionnaire (DEBQ) (van Strien et al., 1986; Cebolla et al., 2014) is a 33-item measure that assesses restrained eating, emotional eating, and external eating triggered by environmental or emotional cues.

*Change scores across time in Cognitive measures at pre-treatment (week 2), post-treatment (week 6) and follow-up (week 18)*

a) **Motor inhibition**. The online food specific Go/NoGo task (Teslovich et al., 2014) will be used to measure motor response inhibition. Participants will be instructed to press the spacebar when a Go signal appears and to withhold their response when a No-Go signal appears. The stimulus set consists of 30 full-color pictures of commonly used high-calorie (*n* = 8) and low-calorie (*n* = 7) foods, as well as neutral items of daily life. The task includes four separate blocks of high-calorie food Go with neutral No-Go; low-calorie food with neutral No-Go; neutral Go with high-calorie food No-Go; and neutral Go with low-calorie food. Reaction time and commission errors for both Go and No-go stimuli will be recorded and analyzed by stimulus type (high-calorie, low-calorie foods and items of daily life). Although inhibitory control will be also assessed with another task (fMRI food specific Go/NoGo task), we decided to include this measure because fMRI will not be performed at follow-up, and a comparable task across all three time points is needed to enable longitudinal analyses of inhibitory control.
b) **Cognitive inhibition**. The Food Stroop Task (Davidson and Wright, 2002) will be used to measure cognitive inhibition, operationalized as the ability to suppress the interference from the emotional meaning of food-related words during color-naming performance. Participants are asked to name the ink color in which a word is printed while ignoring the word itself (which describes a different color). Increased response latency is interpreted as greater interference from task-irrelevant semantic information.
c) **Inhibition and activation systems**. The Punishment Sensitivity and Reward Sensitivity Questionnaire (PSRSQ) (Torrubia et al., 2001) consist of 48 dichotomous items (Yes/No) and includes two subscales of 24 items each: Punishment Sensitivity (related to the inhibition behavioral system) and Reward Sensitivity (related to the behavioral activation system), based on Gray's reinforcement sensitivity theory.
d) **Delay of gratification**. The Food Delay Discounting Task (DD) (Kirby et al., 1999) will be used to assess the preference for immediate vs. delayed rewards. Participants choose between smaller immediate rewards and larger delayed rewards across varying time intervals. The discounting parameter k will be used as the outcome measure.
e) **Self-reported impulsivity**. The Impulsive Behavior Scale (UPPS-P) (Verdejo-García et al., 2010; Cándido Ortiz et al., 2012) evaluates five personality factors that can trigger impulsive behaviors: negative and positive urgency, lack of premeditation, lack of perseverance and sensation seeking.
f) **Working Memory (WM)**. The N-back Task (Kirchner, 1958) evaluates WM updating and interference control. Participants see a series of visual stimuli and indicate whether each stimulus matches the one presented 1, 2 or 3 trials earlier (depending on the block). This task requires continuous updating, storage and removal of irrelevant stimuli from WM. Performance is measured using an ‘efficiency score' that integrates accuracy and reaction time.
g) **Cognitive Flexibility**. The Modified Card Sorting Test (MCST) (Pino et al., 2016) is a measure of set-shifting and abstract reasoning. Participants are instructed to sort response cards according to feedback (correct/incorrect) based on changing rules. The test consists of two packs of 24 response cards in each, and four stimulus cards, varying in shape, color, and number.
h) **Decision making**. The Iowa Gambling Task (IGT) (Bechara et al., 1994) assesses real-world decision-making under uncertainty in a lab setting. Participants are asked to maximize profit over 100 trials by selecting cards from four decks. Decks A and B are disadvantageous and risky (high immediate gains but greater long-term losses), and decks C and D are advantageous (smaller immediate gains but better long-term outcomes). Successful performance reflects the ability to favor long-term over short-term reward.

*Change scores across time in Motivation to change assessed at Pre-treatment (week 2), post-treatment (week 5) and follow-up (week 17)*.

a) **Motivation to change**. The Stages of Change Readiness and Treatment Eagerness Scale (SOCRATES 00) (Vieira Da Silva et al., 2020), adapted for individuals with maladaptive eating behaviors, will be used to assess motivations to change. This self-report instrument consists of 18 items and evaluates individuals' readiness to initiate change in the context of problematic food use.

#### 2.4.4 Change across time in biological samples collection at pre-treatment (week 2), post-treatment (week 6) and follow-up (week 18)

Biological samples will be collected and deep-frozen in CIMCYC research freezers until batch analyses are performed at the end of the project. For blood analyses, 10 mL of fasting blood will be drawn via venepuncture, centrifuged to separate plasma, and stored at −80 °C until processing.

a) **Hormone Levels**. Fasting blood samples will be analyzed to quantify concentrations (pg/mL) of estradiol, progesterone, cortisol, leptin, adiponectin, TSH, thyroxine, triiodothyronine, ghrelin, glucagon and GLP-1.
b) **Glucose and triglycerides levels**. Fasting blood samples will be used to determine serum levels of glucose and triglycerides (mg/dL).
c) **Insulin Levels**. Fasting blood samples will be used to determine insulin concentration (U/mL).
d) **Inflammatory Parameters**. Plasma concentration of interleukin-6 (IL-6; pg/mL), C-reactive protein (CRP; mg/L) and tumor necrosis factor-alpha (TNF-α; pg/mL) will be determined.
e) **Satiety Markers**. Plasma levels of peptide YY (PYY 3–36; pg/mL) will be measured as a marker of satiety signaling.
f) **Genetic Analyses**. DNA will be extracted from the buffy coat obtained during centrifugation. Genetic analyses will include targeted sequencing candidate genes and genome-wide association studies (GWAS) to identify potentially associated with binge eating, including ZFP36, GAD2 (chromosome 10p12), and Neuromedin β. Whole-exome sequencing and DNA methylation analysis will also be conducted.
g) **Oral Microbiota**. Oral swabs will be collected from both cheeks using sterile swabs and stored at −80 °C. Microbiome composition will be determined by taxonomic profiling.
h) **Gut Microbiota**. Participants will provide fecal samples. A 1.5 g aliquot from the surface layer will be stored at −80 °C for subsequent analyses of microbial taxa composition.
**Proteomics**. Proteomics analyses will be performed to determine circulating protein concentration (mg/mL). Blood samples will be centrifuged and plasma stored at −80 °C until batch processing.

#### 2.4.5 Descriptive and screening measures (pre-treatment week 2)

a) **Sociodemographic questionnaire** (age, education, sex, socioeconomic variables) and clinical variables to consider exclusion and inclusion criteria.
b) **BMI**. Weight for BMI calculation (kg/m2) will be obtained with a digital weight (TANITA Corporation of America Inc., Arlington Heights, IL), and height with a measuring rod (SECA Tape Measure 206). This will be measured to ensure BMI within the established inclusion criteria.
c) **Depression Symptoms**. Participants with a score above 29 in the Beck Depression Inventory (BDI-II) (Sanz et al., 2003), indicating severe symptoms, will be excluded.
d) **Anxiety**. Participants with anxiety scores greater than 8 on the Anxiety subscale of the DASS-21 (Daza et al., 2002), indicating severe symptoms, will be excluded.
e) **Eating Disorders**. Questionnaire on Eating and Weight Patterns-5 (QEWP-5) (Yanovski et al., 2015) will be used to exclude people with bulimia. The questionnaire is adapted to DSM-5 criteria.
f) **Binge eating**. The Binge Eating Scale (BES) (Gormally et al., 1982) will be used to ensure people with binge eating problems are included.
g) **fMRI and rTMS Safety Questionnaires**. Standardized safety questionnaires specific to each technique, as approved by the research center, will be administered to collect relevant information from participants prior to undergoing fMRI or rTMS procedures.

#### 2.4.6 Measures to calculate cost effectiveness, cost utility, and budget impact analysis. Administered pre-treatment (week 2), post-treatment (week 5) and follow-up (week 17)

a) **Quality of Life**. The SF-36 Quality of Life Questionnaire will be used to estimate utility values, applying the tariff validated for the Spanish population (Alonso et al., 1995).
b) **Economic Evaluation**. Quality-adjusted life years (QALY), derived from the SF-36 responses, will serve as the primary outcomes in the cost-utility analysis. QALYs combine both the quantity and quality of life and represent the most commonly used metric in health economic evaluations.
c) **Binge eating symptoms**. The Binge Eating Scale (BES) (Gormally et al., 1982) will be used to assess clinical improvements in binge eating behaviors and to inform cost-effectiveness calculations of the intervention.
d) **Food Craving**. The Food Cravings Questionnaire (FCQ-t-r) (Innamorati et al., 2015) will be included as a clinical outcome to assess intervention effectiveness in the cost-effectiveness analysis.
e) **Health Resource Utilization**. The Health Resources Questionnaire will capture participant's use of healthcare services, including visits to primary care, emergency services, hospital admissions, and medication use.
f) **Personnel Costs—iTBS Sessions**. The cost of personnel for iTBS sessions will be calculated based on the time allocated by a Clinical Psychology Specialist (FEA), estimated at 3 min per session across 10 days. Hourly cost will be based on official remuneration tables from the Andalusian Public Health System.
g) **Personnel Costs—Cognitive Training Sessions**. Similarly, the time devoted by a Clinical Psychology Specialist (FEA) to deliver each cognitive training session—estimated at 10 min per session over 10 days—will be calculated according to standard health service rates.
h) **Equipment cost**. The costs associated with the use of fMRI and iTBS equipment for assessment and stimulation sessions will be included in the overall economic evaluation.

### 2.5 Procedure and interventions

Information meetings and psychological assessment sessions will be conducted online. In contrast, biological assessment, fMRI, and neurocognitive interventions will take place on-site. Participation will be organized in small groups of 5–6 individuals. Eligible participants will attend an information session where they will receive written and verbal details about the project. Informed consent will be obtained for assessments, interventions, and for the collection and use of personal data and biological specimens. Participants will then be randomly assigned to one of the three study groups and assigned an anonymous identification code to ensure confidentiality.

All three groups will complete the full assessment protocol. All participants will receive inhibitory control training; the key difference between groups will be the site of iTBS stimulation: left dlPFC, left vmPFC, or control site stimulation applied to the vertex. All procedures will be administered by psychologists, except for the collection of biological samples, which will be performed by a nurse.

At the end of the study, if any of the active stimulation conditions demonstrate efficacy, the intervention will be offered to participants in the other groups. All participants will receive an individualized report with selected results and information about their assigned experimental group after the follow-up assessment.

**Figure 1:**
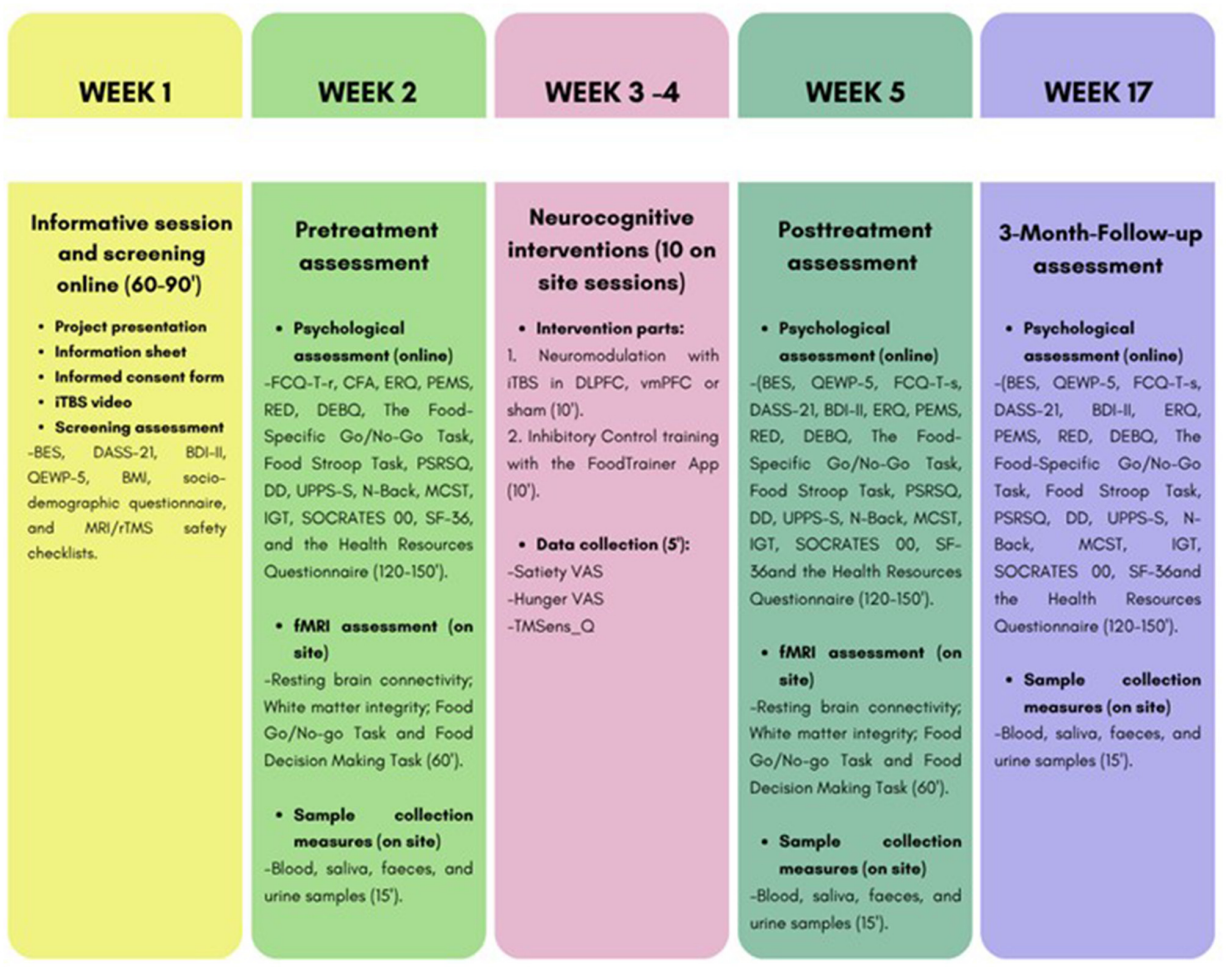
Complete procedure of the protocol.

**Figure 2:**
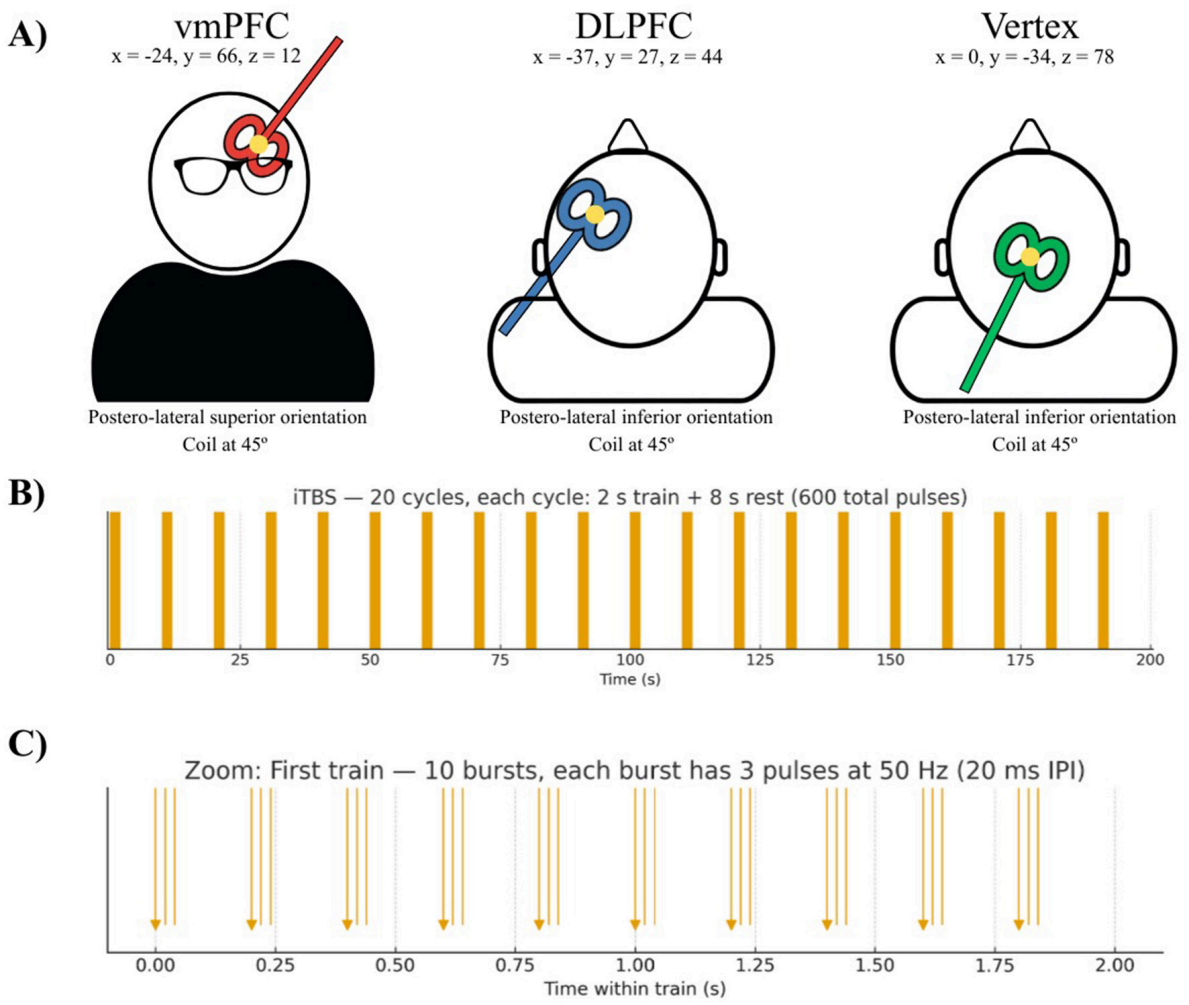
Neuromodulation with iTBS. **(A)** Intermittent theta burst stimulation (iTBS) over the left ventromedial prefrontal cortex (vmPFC-red), left dorsolateral prefrontal cortex (DLPFC-blue) or the vertex (Cz-green) as control site with their respective MNI coordinates, coil and handle orientation. The yellow dot indicates the current exit point. **(B)** Full stimulation timeline showing the 20 iTBS cycles delivered over 200 s. Each cycle consists of a 2-s stimulation train followed by an 8-s rest period, totalling 600 pulses. Vertical lines represent individual TMS pulses. **(C)** Zoomed view of the first 2-s train. The train contains 10 bursts; each composed of 3 pulses delivered at 50 Hz (inter-pulse interval: 20 ms). Bursts are presented every 200 ms. This panel illustrates the characteristic triplet-burst structure of iTBS.

The whole 17-week-procedure consists of the following (See Figure 1):

Informative session and screening (week 1, session 1): This group session is designed to ensure that all participants fully understand the rationale of the intervention. Participants will be informed about the objectives, theoretical framework, and study procedure. If after the explanation they decide to participate, written informed consent and screening measures will be obtained during this session too (measures (BES, DASS-21, BDI-II, QEWP-5, BMI, socio-demographic questionnaire, and MRI/rTMS safety checklists).
Pretreatment assessment (week 2, sessions 2, 3, 4, and 5): Psychological assessment will be conducted online over the course of 1 week. For session 2, different questionnaires are distributed via email, each linked to a separate LimeSurvey URL (FCQ-T-r, CFA, ERQ, PEMS, RED, DEBQ, PSRSQ, DD, UPPS-S, SOCRATES 00, SF-36, and the Health Resources Questionnaire). This procedure will allow participants to complete the 60–90 min session in several sittings at their own pace to minimize fatigue. In session 3, the neuropsychological battery will be administered through the Millisecond platform (The Food-Specific Go/No-Go Task, Food Stroop Task, N-Back, MCST and IGT). Neuropsychological tasks must be completed in a single uninterrupted session of 60 min, at a time chosen by the participant within that week. Participants receive clear instructions on the importance of performing all assessments 2 h after their last meal. Thus, the entire psychological and neuropsychological assessment conducted via email has an estimated total duration of two and a half hours and its duration remains consistent across all evaluation time points. The research team monitors adherence throughout the assessment phase, sending reminders when necessary and verifying that all instruments are properly completed before participants begin the intervention. This flexible design reduces participant burden and minimizes the likelihood of missed assessments. Session 4 consists of an individual fMRI session at the CIMCYC lasting approximately 90 min. This will include assessment of Resting-state connectivity, White matter integrity, the Food Go/No-go Task, and the Food Decision Making Task. Session 5 will also take place at the CIMCYC and will be conducted by a nurse. It will involve collection of blood, saliva, urine, and fecal samples. This session will take approximately 20 min.
Neurocognitive intervention sessions (week 3 and 4, sessions 6–15): The intervention phase spans 2 weeks, during which participants will attend five individual sessions per week (total 10 sessions). Each session will last approximately 25 min. Stimulation parameters follow the iTBS protocols previously validated for individuals with disordered eating [based on (Barone et al., 2023)] and adhere to international safety guidelines (Rossi et al., 2009). Each session consists of two parts:

Part 1: Neuromodulation with iTBS (dlPFC, vmPFC or vertex) (3 min). The complete iTBS procedure lasts approximately 10 min, including 3 min of stimulation and preparation time. Specifically:

A. Target localization. The stimulation site will be determined using the T1- weighted structural MRI images processed via *Brainsight neuronavigation software*. Group 1: stimulation will target the left dlPFC area (MNI coordinates x = −37, y = 27, z = 44), corresponding to the F3 position of the EEG 10-20 system (Beam et al., 2009). Group 2: will target the left vmPFC (x = −24, y = 66, z = 12). Control group: the stimulation control site will be the vertex (x = 0, y = −34, z = 78). For all three targets, the coil will be held tangentially to the scalp and oriented at 45° from the midline, with a posterol-lateral inferior handle orientation for dlPFC and vertex sites, and postero-lateral superior for the vmPFC (see Figure 2). Previous studies showed that the vertex as an active control site placing the coil at ~45° relative to the midsagittal line with a postero-lateral handle orientation does not elicit site-specific behavioral modulation (Martín-Signes et al., 2024; Martín-Arévalo et al., 2019). This type of control site replicates the sensory experience of active stimulation (Boer et al., 2021) and ensures spatial specificity, thereby strengthening both the credibility of control site stimulation and the internal validity of the trial (Picazio et al., 2024). To preserve double-blind conditions, the researcher administering the stimulation will be different from those conducting the cognitive training and assessments.
To ensure comparability across conditions, the TMSens-Q will be applied to confirm similar levels of discomfort and peripheral sensations. Thus, although the handle direction differs slightly between vmPFC and dlPFC/vertex, all three sites are ultimately stimulated with empirically validated orientations that ensure both tolerability and consistency across participants.
B. Stimulation parameters. iTBS will be delivered using *a Magstim Rapid* ^∧^*2 system and a D70alpha figure-of-eight coil*. Parameters are: 50 Hz frequency, 3 pulses per bust; 10 bursts per train; 2-s train duration; 8-s inter-train interval; 20 cycles (total 600 pulses); 5 Hz burst rate.
C. Stimulation intensity. Consistent with (Barone et al., 2023) iTBS will be delivered using a Magstim Rapid^2^ stimulator and a D70alpha figure-of-eight coil at a fixed intensity of 30% of the maximum stimulator output (MSO) and kept consistent across participants, as previous evidence suggested that adjusting intensity to individual motor thresholds does not necessarily enhance the effects (Kaminski et al., 2011). Importantly, the Rapid^2^ system imposes a technical ceiling around 30% MSO for high-frequency patterned protocols due to its power-supply and capacitor-recharge architecture, a constraint explicitly described in prior TBS studies using this device and identical parameters. Despite this conservative dosing limit, both (Barone et al., 2023)and (Vékony et al., 2018) reported significant changes in clinical and cognitive outcomes using the same intensity. Furthermore, a recent clinical-trial protocol employing the same device family has adopted an even lower fixed intensity [25% MSO; (Stevens et al., 2024)], illustrating current trends toward device-constrained but well-tolerated iTBS dosing. Although the 30% MSO limit represents a potential methodological limitation—since higher intensities might theoretically elicit stronger neuromodulatory effects—the present protocol aligns with the only available clinical precedent in this population and ensures safety, tolerability, and methodological reproducibility.

Part 2: Inhibitory Control Training for 10 min. Immediately after stimulation, participants will complete the training with the FoodT app (Lawrence et al., 2015), which is based on the Food Go/No-Go task described earlier. This mobile task requires participants to tap the screen when Go signals are presented and inhibit response when No-Go signals appear. The training version differs from the evaluation so that healthy food images are always paired with Go signals, while unhealthy food images are consistently paired with No-go signals. Neutral non-food images remain randomly paired (50/50) with Go or No-go signals.

Immediately after each neurocognitive session, participants will report their satiety and hunger levels using a 0–100 visual analog scale (VAS) and the TMSens_Q developed to report secondary effects following TMS application (Giustiniani et al., 2022). Intervention sessions will be scheduled in morning and afternoon blocks, with each participant consistently attending at the same time within their selected time block. All participants will be instructed to have their last meal no later than 2 h prior to each intervention session. In addition, before and after every session, they will be asked whether they have experienced any unusual sensations or discomfort potentially related to the procedure; this information will be recorded in the study database. All sessions will be supervised by the study nurse, who will be present to intervene in the event of any adverse effects related to the intervention.

Posttreatment assessment (week 5, sessions 16, 17 and 18). Participants will repeat the whole initial evaluation in session 16 (BES, QEWP-5, FCQ-T-s, DASS-21, BDI-II, ERQ, PEMS, RED, DEBQ, The Food-Specific Go/No-Go Task, Food Stroop Task, PSRSQ, DD, UPPS-S, N-Back, MCST, IGT, SOCRATES 00, SF-36and the Health Resources Questionnaire). Participants will undergo the fMRI protocol in session 17 and the biological measures will be collected in session 18, following the same procedure as in week 2.
3-Month-Follow-up assessment (week 17, session 19 and 20): 3 months after the last intervention session, participants will be contacted to complete a follow-up assessment. In session 19, they will repeat the psychological administered previously (BES, QEWP-5, FCQ-T-s, DASS-21, BDI-II, ERQ, PEMS, RED, DEBQ, The Food-Specific Go/No-Go Task, Food Stroop Task, PSRSQ, DD, UPPS-S, N-Back, MCST, IGT, SOCRATES 00, SF-36, and the Health Resources Questionnaire). In Session 20, biological data will be collected using the same procedures as in previous assessments.

### 2.6 Compliance, adherence, problems, and solutions

Several strategies will be implemented to enhance adherence to the intervention protocols throughout the study. WhatsApp messages will be used to deliver alerts and reminders, including instructions for upcoming fMRI or iTBS sessions and appointment confirmations. The information session will be conducted in a group format, during which participants will watch a video demonstration of a typical iTBS session. This aims to reduce anxiety and increase familiarity with the procedure. On the first day of iTBS administration, the researcher will provide a detailed explanation of the stimulation device and protocol; for this reason, the duration of the first session will be slightly extended. To support inhibitory control training, accommodation will be made for participants with color vision deficiencies: the Food Trainer app allows visual stimuli to be differentiated by pattern (e.g., continuous vs. dashed lines). If a participant misses a scheduled session, a make-up session will be arranged for the following week. A maximum of three missed intervention sessions may be rescheduled before the participant is considered non-compliant. During the intervention, transient adverse effects related to iTBS (e.g., headache, fatigue, local scalp discomfort) may occur. Participants will be informed in advance, and all sessions will be conducted under on-site clinical supervision by a registered nurse. Any adverse events or observations reported by staff or participants will be documented in a dedicated database and managed according to predefined procedures and complete TMSens_Q (Giustiniani et al., 2022). The intervention team will hold current certification in basic life support and automated external defibrillator use. The study will adhere to international safety recommendations for TMS in research and clinical practice (Rossi et al., 2009, 2021). To support adherence to the inhibitory-control training, sessions will be brief (approximately 10 min) and followed by two brief oral questions assessing satiety and craving. During the posttreatment assessment, participants will complete a debriefing questionnaire to gather feedback on their experience, including any difficulties related to adherence and engagement or perceived burden, as well as a blinding assessment to evaluate the success of the masking procedure. Finally, at the end of the follow-up period, each participant will receive a personalized report summarizing their main individual results.

Adherence to the intervention will be monitored by recording attendance at all scheduled sessions.

### 2.7 Statistical methods

Inferential statistics will be applied in accordance with the characteristics of the data obtained, including distribution, scale of measurement, and other relevant properties. All analyses will be aligned with the study's hypotheses.

Objectives 1 and 2 will be addressed using repeated measures mixed models (Gueorguieva and Krystal, 2004), with BE frequency, craving, cognitive performance, food intake, and related behaviors as dependent variables. The independent variable will be the intervention condition: iTBS of the dlPFC combined with inhibitory control training vs. vertex iTBS (objective 1); iTBS of the vmPFC combined with inhibitory control training vs. vertex iTBS (objective 2). Additionally, a planned comparison of both active interventions (dlPFC and vmPFC) vs. vertex will be conducted. Effect sizes will be calculated for both between- and within-group comparisons. Mediation and moderation analysis will assess the influence of baseline characteristics—such as cognition, emotional symptoms, emotional eating, motivation, clinical variables, and adherence to diet and physical exercise—on treatment outcomes. Subgroup analyses will also be conducted to explore differential effects based on sex and BE severity (subthreshold vs. full disorder).

Objective 3 will be examined by analyzing fMRI data following pre-processing using SPM12 (Statistical Parametric Mapping, version 12) in MATLAB R2018a. Resting-state fMRI will be analyzed using two methods: Independent Component Analysis (ICA) to examine alterations in large-scale brain networks, and Seed-based connectivity analyses focusing on target regions (dlPFC and vmPFC). For the two task-based paradigms, psycho-physiological interaction (PPI) analysis will explore functional activity and connectivity related to food evaluation and decision-making. All analyses will be performed in SPM12 (Statistical Parametric Mapping, 2007). DTI images will be analyzed using the FMRIB Software Library (FSL) to extract measures of white matter integrity, including fractional anisotropy (FA) and apparent diffusion coefficient (ADC).

To address objective 4, exploratory analyses will be conducted on biological samples (plasma, genetics, proteomics and microbiota). Correlations and regression analysis will be used to explore conducted association between biological parameters and both neuropsychological and neuroimaging outcomes.

For objective 5, incremental cost-effectiveness (ICER) and incremental cost-utility (ICUR) ratios will be calculated based on direct healthcare costs and outcomes (e.g., changes in BE symptoms and QALYs), following CHEERS guidelines (Husereau et al., 2013) and Spanish health economic evaluation standards (López-Bastida et al., 2010). If baseline utility scores differ significantly, results will be adjusted via bivariate regression. Deterministic sensitivity analyses (DSA) will assess the impact on individual parameters. Probabilistic sensitivity analyses (PSA) using non-parametric bootstrapping (1,000 iterations) will evaluate ICUR uncertainty, with results presented as cost-effectiveness plans and acceptability curves. The accepted cost-effectiveness threshold in Spain (€20,000/QALY) will be used as reference (Vallejo-Torres et al., 2018). Budget impact analysis will estimate the costs of implementing iTBS and cognitive training across the public healthcare system, considering prevalent-based population estimates. Univariate and multivariate SA will examine how variations in unit cost or patient volume influence cost-effectiveness.

All analyses will follow both intention-to-treat (ITT) and per protocol (PP) principles. Appropriate corrections will be applied to adjust for multiple comparisons. Missing data will be managed using last observation carried forward (LOCF) imputation.

The success of the masking procedure in the blinding assessment will be evaluated by comparing the proportion of correct guesses against chance using a chi-square test and by computing the James' Blinding Index (James et al., 1996). Confidence ratings will be analyzed descriptively.

## 3 Expected results

It is hypothesized that both stimulation conditions will lead to improvements in food related decision-making and reductions in the frequency and intensity of binge eating episodes. Furthermore, the combination of iTBS and inhibitory control training is expected to produce favorable outcomes in terms of cost-effectiveness and cost-utility. Expected outcomes include reductions in BE symptoms and food cravings, as well as changes in brain connectivity and activation patterns during rest and food-related tasks. Improvements are also anticipated in eating behavior. Additionally, the intervention is expected to alleviate emotional symptoms and maladaptive eating patterns—such as depression, anxiety, difficulties in emotional regulation, emotional eating, and reward-driven eating. Cognitive domains hypothesized to improve include motor and cognitive inhibition, delay discounting, impulsivity, working memory, cognitive flexibility, and decision-making. Biological changes—such as alterations in plasma markers and microbiota composition—are also anticipated. Finally, positive results in economic evaluations (cost-effectiveness and cost-utility) are expected for the combined intervention.

The results of the clinical trial will be disseminated through conferences and peer-reviewed journals, according to open science principles.

## 4 Discussion

This double-blind, randomized, controlled trial with parallel groups aims to evaluate the efficacy of intermittent theta burst stimulation (iTBS) targeting either the dorsolateral or ventromedial prefrontal cortex, combined with inhibitory control training, as an intervention for binge eating. The objective is to assess its impact on brain, behavioral, emotional, cognitive and biological outcomes in individuals with binge eating behavior. The study entails several challenges, including the recruitment of 150 participants capable of attending multiple in-person sessions at the CIMCYC several times, and the evaluation of medium-term outcomes (three-month follow-up) across three intervention conditions. On the other hand, the access to neuronavigation systems such as Brainsight may not be uniformly available across research and clinical environments, which could affect the straightforward replication of protocols relying on this technology. Nevertheless, while neuronavigation is indispensable for the present experimental comparison, the protocol can be readily adapted to broader research contexts, and its outcomes remain relevant for clinical and translational applications employing validated coordinate-based procedures.
